# Reactive Oxygen Species Donors Increase the Responsiveness of Dorsal Horn Neurons and Induce Mechanical Hyperalgesia in Rats

**DOI:** 10.1155/2015/293423

**Published:** 2015-09-20

**Authors:** Hee Young Kim, Inhyung Lee, Sang Woo Chun, Hee Kee Kim

**Affiliations:** ^1^Department of Neuroscience and Cell Biology, 301 University Boulevard, University of Texas Medical Branch, TX 77555-1069, USA; ^2^Department of Physiology, College of Korean Medicine, Daegu Haany University, Daegu 706-060, Republic of Korea; ^3^Department of Veterinary Clinical Sciences, College of Veterinary Medicine, Seoul National University, 1 Gwanak-ro, Gwanak-gu, Seoul 151-742, Republic of Korea; ^4^Department of Oral Physiology, College of Dentistry, Institute of Wonkwang Biomaterial and Implant, Wonkwang University, 344-2 Shinyong Dong, Iksan 570-749, Republic of Korea; ^5^Department of Pain Medicine, The University of Texas MD Anderson Cancer Center, 1515 Holcombe Boulevard, Houston, TX 77030, USA

## Abstract

Our previous studies suggest that reactive oxygen species (ROS) scavengers have analgesic effect on neuropathic pain through spinal mechanisms in the rat. The studies suggest that superoxide in spinal cord is one of important mediators of persistent pain. To test the hypothesis that increase of superoxide-derived intermediates leads to central sensitization and pain, the effects of an intrathecal injection of chemical ROS donors releasing either OH^∙^, OCl^−^, or H_2_O_2_ were examined on pain behaviors. Following treatment with* t*-BOOH (OH^∙^ donor), dorsal horn neuron responses to mechanical stimuli in normal rats and the changes of neuronal excitability were explored on substantia gelatinosa (SG) neurons using whole-cell patch clamping recordings. Intrathecal administration of* t*-BOOH or NaOCl (OCl^−^ donor), but not H_2_O_2_, significantly decreased mechanical thresholds of hind paws. The responses of wide dynamic range neurons to mechanical stimuli increased after a local application of* t*-BOOH. The* t*-BOOH increased the frequency and the amplitude of excitatory postsynaptic potentials, depolarized membrane potential in SG neurons, and increased the frequency of action potentials evoked by depolarizing current pulses. These results suggest that elevated ROS, especially OH^∙^, in the spinal cord sensitized dorsal horn neurons and produced hyperalgesia in normal rats.

## 1. Introduction

Reactive oxygen species (ROS) are generated as part of normal cell metabolism and serve both normal physiological and pathophysiological functions [1, 2]. The many types of ROS include superoxide radicals (O_2_^∙−^), hydroxyl radicals (OH^∙^), hydrogen peroxide (H_2_O_2_), nitric oxide (NO), and peroxynitrite [2, 3]. The major source of ROS in the central nervous system is the electron transport chain in the inner membranes of mitochondria that produces adenosine triphosphate. Leakage of electrons during electron transport produces the superoxide anion (O_2_^∙−^), which transforms OH^∙^ in the presence of transition metals such as free iron. The overproduction of ROS results in lipid peroxidation, protein oxidation, and nucleic acid oxidation [4].

ROS have been implicated in the pathogenesis of various diseases, including rheumatoid arthritis, asthma, inflammatory bowel disease, atherosclerosis, and Alzheimer disease. Previous studies suggested that ROS are critically involved in various pain conditions, including neuropathic and inflammatory pain. ROS scavengers such as superoxide dismutase mimetics [5], phenyl N-*t*-butylnitrone [6, 7], 5,5-dimethyl-1-pyrroline-N-oxide [6], and vitamin E [8] reduce hyperalgesic behaviors in several rat models of pain. The main action site for ROS in neuropathic [6, 8] and capsaicin-induced pain [7, 9] is the spinal cord. Also, peripheral nerve injury increases the production of ROS in the spinal cord in persistent pain conditions [10]. Those findings suggest that ROS in the spinal cord are critically involved in neuropathic and inflammatory pain. Furthermore, our previous studies have suggested that increased production of the primary ROS, O_2_^∙−^, from mitochondria mediates sensitization of spinal dorsal horn neurons and thus persistent pain [7, 11]. In brief, O_2_^∙−^ scavengers reduce persistent pain, dorsal horn neuron hyperexcitability [7], and spinal long-term potentiation [11]. The level of mitochondrial O_2_^∙−^ dismutase determines the level of central sensitization and thus hyperalgesia [12]. While it is generally accepted that O_2_^∙−^ do not diffuse across membranes [13], it is not clear how the O_2_^∙−^ formed in mitochondria diffuse into the cytoplasm and mediate spinal neuronal plasticity and pain. O_2_^∙−^ in mitochondria are rapidly converted to membrane permeable hydrogen peroxide (H_2_O_2_) by superoxide dismutase, are diffused in cells, and can be sequentially converted to highly reactive oxidants, such as OH^•^ and hypochlorite (OCl^−^) [14]. Here, we hypothesized that an increase in superoxide-derived intermediates in spinal dorsal horn neurons leads to central sensitization and hyperalgesia.

In the present study, we examined if artificial increases in possible superoxide intermediates—OH^∙^, OCl^−^, and H_2_O_2_—in the spinal dorsal horn produce pain behaviors in normal rats. To further explore the role of OH^∙^ in pain, we used* in vivo* extracellular recordings to examine if* tert*-butyl hydroperoxide (*t*-BOOH, an OH^∙^ donor) increases the excitability of wide dynamic range (WDR) neurons in the spinal dorsal horn, and we used* in vitro* intracellular recordings to examine if* t*-BOOH produces changes in membrane excitability in substantia gelatinosa (SG) neurons in spinal cord slice preparations.

## 2. Materials and Methods

### 2.1. Experimental Animals

We used male Sprague-Dawley rats (Harlan Sprague-Dawley Co., Houston, TX, USA) for the experiments. The rats were housed under a 12/12-hour reversed light-dark cycle (dark cycle, 8:00 A.M.–8:00 P.M.) for at least 1 week before any experiments. All experiments were carried out in accordance with the National Institute of Health's Guide for the Care and Use of Laboratory Animals, and the animal use protocol was approved by the University of Texas Medical Branch Institutional Animal Care and Use Committee.

### 2.2. Behavioral Test

#### 2.2.1. Intrathecal Catheterization

Intrathecal catheters were implanted into the lumbar enlargement as described previously [6, 8]. Briefly, adult rats (200–350 g) were anesthetized with isoflurane (3% for induction and 2% for maintenance) in the flow of oxygen, and then a posterior midline incision was made from the T11 vertebra to L1. The posterior articular process and lamina of the T12 vertebra were removed with a pair of rongeurs to expose the spinal meninges. A small nick was made on the dura mater, and a prepared catheter (sterilized tubing filled with saline; PE10, Becton Dickinson) was inserted into the intrathecal space. The catheter was gently guided caudally until the tip reached the level of the lumbar enlargement of the spinal cord (approximately 1 cm caudal to the initial insert point). The remaining part of the tubing was connected with PE50 tubing and fed subcutaneously to the midthoracic level with anchors to the muscles at multiple sites in order to expose the tip to the dorsal midline position. The outside tip of the tubing was sealed, and the incision was closed. After full recovery from anesthesia, the rats were returned to their cages and housed individually for 1 week. Catheters were flushed with 10 *μ*L of sterile saline 3 days after catheterization to maintain patency. This experiment excluded rats showing dragging of hind paws or 5% loss of body weight 7 days after catheterization. The position of the intrathecal catheter was checked after the animals were euthanized at the end of the experiment.

#### 2.2.2. Intrathecal Application of ROS Donors

Either* t*-BOOH or NaOCl (both from Sigma Chemical Company, St. Louis, MO, USA) was injected through an intrathecal catheter for 2-3 min while the animals were conscious at least 1 week after lumbar catheterization. The OH^∙^ donor* t*-BOOH was administered at 11, 28, or 55 *μ*mol in a volume of 15 *μ*L. The hypochlorite donor NaOCl was injected at 134 *μ*mol in 15 *μ*L. Control rats were treated with 15 *μ*L of sterile 0.9% saline. In addition, H_2_O_2_ was directly injected into the intervertebral space between the L5 and L6 vertebras under light isoflurane anesthesia at 15, 44, 74, or 148 *μ*mol in 50 *μ*L.

#### 2.2.3. Behavioral Testing for Mechanical Thresholds

Behavioral tests were conducted to measure the 50% foot mechanical thresholds in response to mechanical stimuli applied to both the left and the right hind paws under blind conditions. We recorded the mechanical threshold for the paw that was more sensitive to stimuli. For testing, each animal was placed in a plastic chamber (8.5 × 8.5 × 28 cm) that was placed on top of a mesh screen, and mechanical stimuli were applied to the plantar surface of one hind paw with von Frey monofilaments from underneath. Thresholds were determined by the up-down method [15] using von Frey monofilaments 4.10, 4.31, 4.52, 4.74, 4.92, and 5.16 (equivalent to 1.26, 2.04, 3.31, 5.50, 8.32, and 14.45 g, resp.). von Frey filaments were applied perpendicularly to the most sensitive area of the plantar surface—the proximal portion and base of the 2nd, 3rd, or 4th toe—with sufficient force to bend the filament slightly for 2 to 3 s. An abrupt withdrawal of the foot during stimulation or immediately after stimulus removal was counted as a positive response. The first stimulus was always initiated with the 4.74 filament. If there was a positive response, the next-lower-strength filament was used, and if not, the next-higher-strength size filament was applied. This testing pattern was continued until we had recorded responses to six von Frey stimuli counting from the first change of response (i.e., a positive response to a stimulus after a negative response to the first stimulus or a negative response to a stimulus after a positive response to the first stimulus). The responses were then converted into a 50% threshold value using the formula 10^(*X* + *kd*)^/10^4^, where *X* is the value of the final von Frey filament used in logarithmic units, *k* is the tabular value for positive/negative responses, and *d* is the mean difference between stimuli in logarithmic units (0.22) [16]. When positive or negative responses were still observed at the end of a stimulus session, values of 3.54 or 5.27 were assigned, respectively, by assuming a value of ±0.5 for *k* in these cases. The behavioral data were plotted using a linear scale in von Frey values as well as in grams.

### 2.3. Extracellular Recordings of WDR Neurons in the Spinal Cord

Adult rats (200–350 g) were anesthetized with an intraperitoneal injection of urethane (1.5 g/kg). The trachea was cannulated to provide unobstructed ventilation, and a catheter was inserted into the left external jugular vein. A laminectomy was performed to expose the spinal cord at the T13-L2 vertebral level. The rat was placed in a stereotaxic apparatus, and the spinal cord was bathed in a pool of warm mineral oil. Core body temperature was maintained at 37°C by a controlled heating blanket. The animal was paralyzed with an initial intravenous bolus dose of pancuronium bromide (1 mg/kg) and ventilated artificially to maintain end-tidal CO_2_ between 3.5 and 4.5%. The level of pancuronium was maintained by continuous intravenous infusion (0.4–0.6 mg/kg/h).

An extracellular recording was made for WDR neurons in the dorsal horn. These neurons responded to both innocuous and noxious mechanical stimuli. Cells were searched at the L4 and L5 segments of the spinal cord using a low-impedance (0.4–0.8 MΩ) carbon filament electrode (Kation Scientific, Minneapolis, MN, USA) mounted on an electronic micromanipulator. Brush stimuli were used to search for dorsal horn neurons. WDR neurons with receptive fields located on the plantar surface of the ipsilateral hind paw were recorded extracellularly. Recordings were made only for single neurons whose spike amplitude could be easily discriminated from those of other neurons (at least twice the height). Electrophysiological activity was amplified, displayed on an oscilloscope, and transmitted into a data analysis system (CED 1401, PC, USA) with Spike2 software. Throughout the experiment, spike sizes and configurations were continuously monitored with the use of Spike2 software to confirm that the data were being acquired from the same WDR neuron and that the relationship of the recording electrode to the neuron remained constant.

After the receptive field of a WDR neuron was confirmed, graded mechanical stimuli were applied: soft-brush, nonnoxious (1 or 2 g) von Frey filaments and a noxious (20 g) von Frey filament. All stimuli were applied at a rate of once per second for 10 s with a 10 s interval between stimuli. Background activity was recorded three times before the administration of* t*-BOOH. Ten microliters of a* t*-BOOH solution (1 or 10 *μ*mol) was applied to a small cotton ball around the electrode on the spinal cord. The cotton ball was kept on the spinal cord after application of* t*-BOOH until the end of the experiment. The discharges of WDR neurons were recorded every 30 min after administration of* t*-BOOH. Responses to mechanical stimuli were counted as discharges per second during 10 s of stimulation, and three separate counts were obtained for each animal.

### 2.4. Patch Clamp Recordings of SG Neurons in the Spinal Cord

#### 2.4.1. Spinal Cord Slice Preparation

Sprague-Dawley rats (14–20 days old, 30–55 g) were anesthetized with urethane (1.5 g/kg, intraperitoneally), and a lumbosacral laminectomy was performed. The lumbar spinal cord was rapidly dissected and submerged in ice-cold artificial cerebrospinal fluid (ACSF). The dura and arachnoid membranes and ventral/dorsal roots around the lumbar spinal cord were removed. The spinal cord was mounted on a 752M Vibroslicer (Campden Instruments, Leicestershire, UK) and cut into 300 *μ*m thick transverse slices. The slices were then incubated in ACSF, saturated with 5% CO_2_ in oxygen at 32°C for 1 h, and transferred to a recording chamber, which was continuously perfused with aerated ACSF at a rate of 3-4 mL/min. The ionic composition of the ACSF was 117 mM NaCl, 3.6 mM KCl, 1.2 mM NaH_2_PO_4_, 1.2 mM MgCl_2_, 2.5 mM CaCl_2_, 11 mM glucose, and 25 mM NaHCO_3_ (pH 7.4). A platinum grid was placed on top of the slice to prevent slice movement.

#### 2.4.2. Patch Clamp Recordings

Whole-cell recordings using the patch clamp technique were performed for SG neurons in the lumbar spinal cord. Patch electrodes were made from borosilicate glass capillaries (1.5 mm diameter and 0.25 mm wall thickness) pulled on a P-80 micropipette puller (Sutter Instruments, Novato, CA, USA). When an electrode was filled with the internal solution, the resistance of patch electrodes was 6–8 MΩ. The internal solution was composed of 150 mM potassium gluconate, 5 mM KCl, 0.1 mM ethylene glycol-bis(*β*-aminoethylether)-N,N,N′,N′-tetraacetic acid (EGTA), 10 mM N-2-hydroxyethylpiperazine-N′-2-ethanesulfonic acid (HEPES), and 5 mM MgATP (pH 7.25). Recordings were made of neurons within the SG, which was visible as a distinct translucent band across the dorsal horn under a microscope (BX51WI, Olympus, Tokyo, Japan). Current-clamp recordings were made using an Axopatch 200B amplifier (Axon Instruments, Foster City, CA, USA). After filtration at 2 KHz using a low-pass filter, data were acquired using a Digidata 1322A interface and pClamp software (version 9.0, Axon Instruments) for subsequent analysis. All recordings were made at room temperature (20–25°C).

After the formation of more than 1 GΩ, whole-cell access was achieved by rupturing the membrane with negative pressure. The resting membrane potential was measured 5–10 min after whole-cell configuration. Only neurons with a resting membrane potential more negative than −50 mV were used. Excitatory postsynaptic potentials (EPSPs) were analyzed with the Mini Analysis program (version 6.0, Synaptosoft, Decatur, GA, USA). The frequency of EPSPs was determined by setting a detection threshold level (1.0–2.0 mV). The excitability of SG neurons was quantified by examining the number of action potentials (spikes/second) evoked in response to a current of 25 pA or 50 pA (1 s duration for adapting-firing neurons (AFNs) and 3 s duration for tonic-firing neurons (TFNs)) from a holding potential of −60 mV.* t*-BOOH in ACSF was applied to the perfusion bath by a gravity perfusion system (BPS-4, ALA Scientific Instruments, Westbury, NY, USA).

### 2.5. Statistical Analysis

The data were summarized in terms of mean and standard errors of the mean and analyzed using the statistics program SigmaStat (version 3.1, Systat Software, San Jose, CA, USA). Statistical significance (*P* < 0.05) was determined using the Student *t*-test and one- or two-way repeated-measures analyses of variance with one repeated factor followed by the Duncan test.

## 3. Results

### 3.1. Intrathecal* t*-BOOH and NaOCl Produced Transient Pain Behaviors

**Figure 1:**
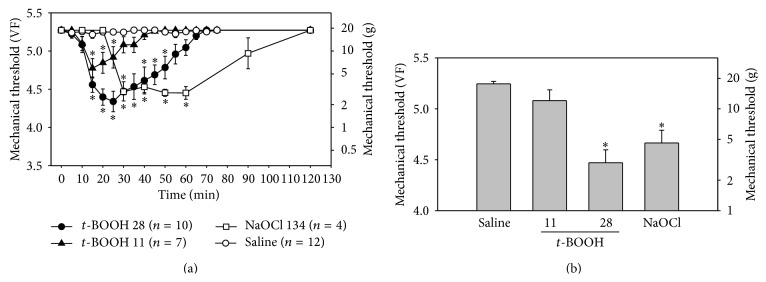
Effects of* t*-BOOH and NaOCl on mechanical thresholds in normal rats. (a) Intrathecal administration of* t*-BOOH (11 or 28 *μ*mol) or NaOCl (134 *μ*mol) temporarily decreased the mechanical threshold in a dose-dependent manner, compared to the mechanical threshold observed with administration of saline. Asterisks indicate values that are significantly different from those for the saline group as determined by a two-way repeated-measures analysis of variance with one repeated factor followed by the Duncan post hoc test (*P* < 0.05). (b) Mechanical thresholds of hind paws after intrathecal administration of* t*-BOOH (11 *μ*mol, *n* = 7; 28 *μ*mol, *n* = 10) or NaOCl (134 *μ*mol, *n* = 4), compared with the mechanical threshold observed with saline, 30 min after injection. Asterisks indicate values that are significantly different from that for the saline group as determined by the Kruskal-Wallis analysis of variance by ranks followed by the Dunn test (*P* < 0.05). The data are mean with standard errors of the mean. VF, von Frey filament sizes.

Intrathecal administration of* t*-BOOH (11 or 28 *μ*mol in 15 *μ*L) decreased the mechanical threshold in a dose-dependent manner (Figure 1(a)). The mechanical threshold started to decrease 10 min after the administration of 28 *μ*mol of* t*-BOOH, reached the lowest point (4.34 ± 0.13 on the von Frey scale) at 25 min, and recovered to near the baseline level at 55 min. The mechanical threshold after the administration of 11 *μ*mol of* t*-BOOH dropped to 4.77 ± 0.13 at 15 min after injection. Because, in a preliminary study, intrathecal administration of 55 *μ*mol of* t*-BOOH induced severe pain behaviors such as body twisting and squeaking right after administration, that dose was not tested further. The 5.5 *μ*mol dose of* t*-BOOH did not influence mechanical thresholds (data not shown).

Intrathecal administration of 134 *μ*mol of NaOCl significantly decreased the mechanical threshold between 25 min and 65 min after administration (Figure 1(a)). At 121 *μ*mol, NaOCl significantly decreased the mechanical threshold between 40 min (4.65 ± 0.13) and 50 min (4.69 ± 0.08). The 100 *μ*mol dose of NaOCl did not have a hyperalgesic effect (data not shown), indicating that NaOCl induces hyperalgesia at a narrow range of doses.

The mechanical threshold observed with either* t*-BOOH at 28 *μ*mol or NaOCl at 134 *μ*mol was significantly lower than that observed with saline at 30 min after administration (Figure 1(b)). The administration of saline had no effect on mechanical thresholds. The areas of the paw that were the most sensitive to von Frey filaments were the base and proximal portions of the 3rd and 4th digits.

Intrathecal administration of H_2_O_2_ at 15, 44, or 74 *μ*mol did not affect the mechanical threshold until 90 min after administration. Because 148 *μ*mol of H_2_O_2_ produced serious side effects and death, doses over 148 *μ*mol were not tested.

Since intrathecal injection of H_2_O_2_ failed to produce pain behaviors in normal rats and since our recent study showed that NaOCl increases the excitability of SG neurons [17], the following experiments focused on the effects of the OH^∙^ donor* t*-BOOH on neuronal excitability.

### 3.2. *t*-BOOH Increased the Responsiveness of Neurons in the Spinal Dorsal Horn

**Figure 2:**
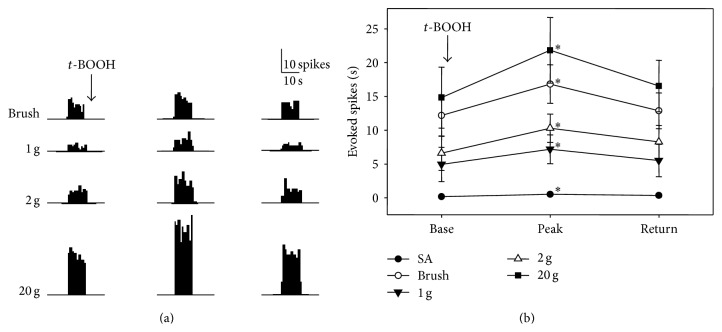
Effect of* t*-BOOH on WDR neuron activities in the spinal dorsal horn of normal rats. (a) The neurons responded well to a variety of mechanical stimuli applied to the receptive field before administration of* t*-BOOH (left column). After local application of 1 *μ*mol of* t*-BOOH in 10 *μ*L to the spinal cord without washout, the discharge rates started to increase, peaked by 60.0 (±14.1) min (center column), and then returned to the baseline levels by 122.1 (±32.1) min (right column). Stimulus duration is indicated by horizontal bars at the bottom. (b) Evoked responses of WDR neurons (*n* = 7) to various stimuli before and after* t*-BOOH application. Asterisks indicate values that are significantly different from the baseline values as determined by a one-way repeated-measures analysis of variance followed by the Duncan test (*P* < 0.05). The data are mean with standard errors of the mean.

Neurons in the spinal dorsal horn responded well to a variety of mechanical stimuli, including the stroking of the skin with a brush and repeated applications of von Frey filaments with weak (1 and 2 g) and strong (20 g) bending forces (Figure 2). Intrathecal* t*-BOOH was applied to a cotton ball around the electrode, and the cotton ball was kept in place until the end of experiment. After application of a* t*-BOOH dose of 1 *μ*mol in 10 *μ*L to the dorsal surface of the spinal cord, the rate of discharges of the seven WDR neurons in response to brushing and von Frey filaments started to increase by 30.0 ± 5.0/min. The rate of discharges peaked at 60.0 ± 14.1 min after administration and returned to near the baseline level at 122.1 ± 32.1 min after administration (Figure 2(a)). After application of a* t*-BOOH dose of 10 *μ*mol in 10 *μ*L to the spinal cord, the rate of discharges of the WDR neurons in response to brushing and von Frey filaments started to dramatically increase by 30 min. At the same time, there was a sudden onset of discharges from the other WDR neurons. Therefore, the 10 *μ*mol dose of* t*-BOOH was not used subsequently.  Figure 2(b) shows the discharge rates after application of 1 *μ*mol of* t*-BOOH. The peak discharge rates after treatment with* t*-BOOH increased by approximately 180% from baseline (183% for brushing, 180% for 1 g, 185% for 2 g, and 171% for 20 g).

### 3.3. *t*-BOOH Increased the Excitability of SG Neurons in Spinal Cord Slices

**Figure 3:**
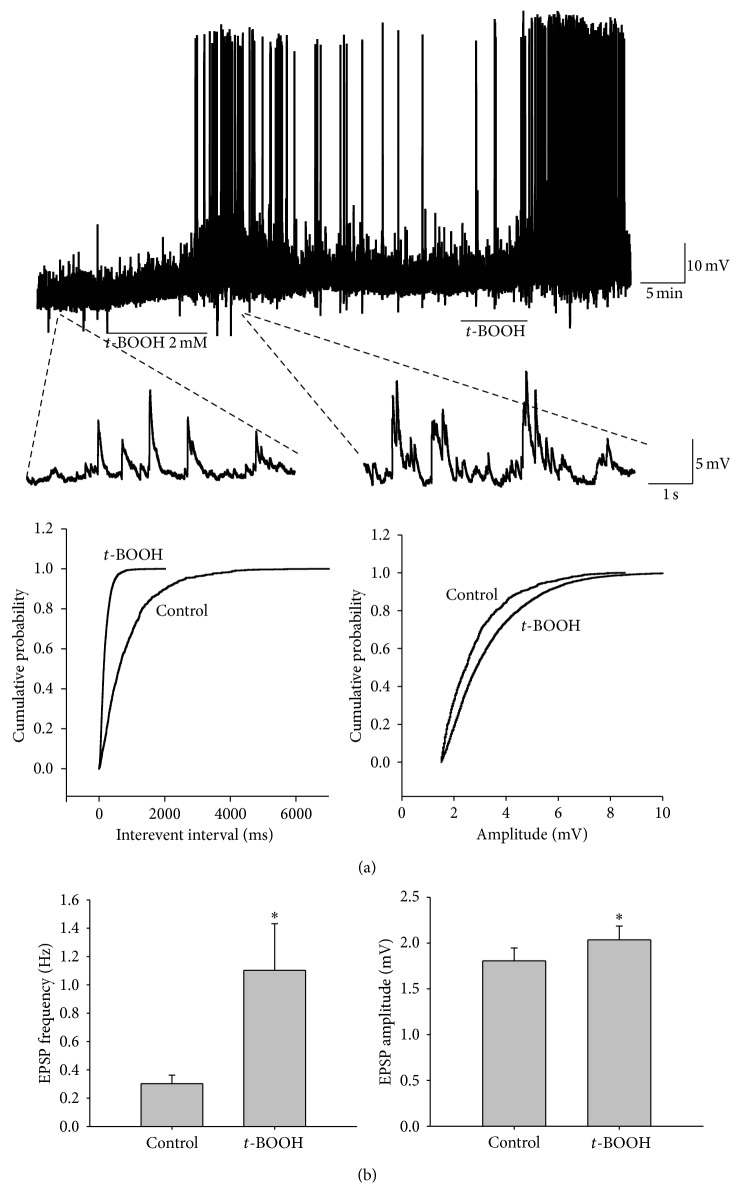
Effects of* t*-BOOH on membrane potential and EPSP in a patch clamp recording of SG neurons. (a) Original amplitude traces of EPSP in an SG neuron show that* t*-BOOH (2 mM, 7 min) increased the amplitude and frequency of EPSPs. The lower traces show EPSP at an expanded time scale. Normalized cumulative distribution analysis of EPSP amplitude and frequency (lower panels) showed that* t*-BOOH caused a significant shift toward higher frequency (left) and amplitude (right) in the neuron. (b) Average EPSP frequency (left) and amplitude (right). After application of* t*-BOOH, both the frequency and the amplitude increased significantly (*P* < 0.05, asterisks) from pre-*t*-BOOH levels as determined by a paired *t*-test. The data are mean with standard errors of the mean.

Because the SG is a major termination site for unmyelinated afferents and plays an important role in pain mechanisms [18], we obtained whole-cell patch clamp recordings to investigate whether* t*-BOOH could change the excitability of SG neurons in spinal cord slices. Only SG neurons with a resting membrane potential more negative than −50 mV were examined. The resting membrane potential was −56.0 ± 1.1 mV (*n* = 26). When the cell was held at −60 mV, the application of* t*-BOOH (2 mM) for 7 min induced a 3.1 ± 0.5 mV depolarization, which was maintained for up to about 30 min after washout and which then recovered to baseline (Figure 3(a)). For measurement of spontaneous EPSPs of SG neurons, the baseline (control) was recorded for at least 10 min in ACSF after the whole-cell recording configuration, and the samples were superfused with* t*-BOOH (2 mM in the ACSF solution) for 7 min and then washed out for 15 min. Superfusion with* t*-BOOH significantly increased EPSP frequency (1.1 ± 0.3 versus 0.3 ± 0.1 Hz, *P* < 0.05) and amplitude (2.1 ± 0.2 versus 1.8 ± 0.1 mV, *P* < 0.05) (*n* = 26) over the baseline (Figure 3). These data suggest that an increase in the level of hydroxyl radicals in the SG neurons affected both presynaptic and postsynaptic mechanisms of excitatory transmission in the rats.

**Figure 4:**
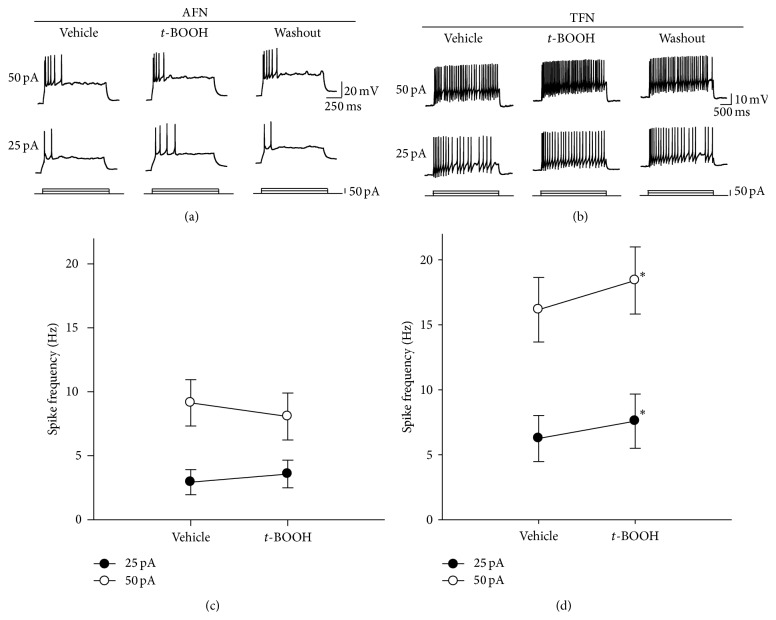
Effects of* t*-BOOH on the excitability of SG neurons in spinal cord slices. (a) Recordings of action potentials generated in an AFN. Action potentials were evoked by direct intracellular injections of current pulses (25 or 50 pA). (b) Changes in the frequency of action potential in a TFN. After* t*-BOOH treatment, the frequency of action potentials increased, compared to that observed with the vehicle treatment. (c) Average action potential firing rate measured by step current pulses after* t*-BOOH treatment in AFNs (*n* = 14). The data are mean with standard errors of the mean. (d) Average action potential firing rate measured by step current pulses after* t*-BOOH treatment in TFNs (*n* = 12). Asterisks indicate values that are significantly different from those for the vehicle control as determined by a paired* t*-test (*P* < 0.05).

Based on action potential (AP) discharge patterns elicited by injections of a current into a cell, SG neurons are classified as (i) AFNs, in which the frequency of an action potential is decreased during membrane depolarization; (ii) TFNs, in which a repetitive action potential is sustained during membrane depolarization; and (iii) delayed-firing neurons (DFN) with delayed-firing onset, as observed previously [19–21]. In preliminary study to determine the threshold at which a current generates an action potential in SG neurons, we stimulated neurons with depolarizing pulses using 2 to 50 pA in increments of 2 pA. An action potential was produced by current pulses of 25.7 ± 2.9 pA and 25.1 ± 4.1 pA in AFNs (*n* = 10) and TFNs (*n* = 9), respectively (data not shown). There was no statistically significant difference in the threshold current of AP generation between AFNs and TFNs. We therefore used two currents, 25 and 50 pA, to compare the numbers of action potentials before and after the application of* t*-BOOH. Mean input resistance was 660 ± 67 MΩ (*n* = 26). In AFNs (*n* = 14), the number of action potentials after the application of* t*-BOOH did not differ from the number before* t*-BOOH treatment (Figures 4(a) and 4(c)). In contrast, after treatment with* t*-BOOH, TFNs (*n* = 12) had significant increases in the frequencies of action potentials: from 6.3 ± 1.8/s to 7.9 ± 2.1/s and from 16.2 ± 2.5/s to 18.4 ± 2.6/s for current pulses of 25 and 50 pA, respectively (Figures 4(b) and 4(d)), indicating that* t*-BOOH increased the excitability of TFNs but not AFNs.

## 4. Discussion

This study showed that intrathecal administration of ROS donors* t*-BOOH (an OH^•^ donor) and NaOCl (an OCl^−^ donor) but not H_2_O_2_ induced mechanical hyperalgesia of the hind paw in a dose-dependent manner in rats. Increased responses of WDR neurons to mechanical stimuli were also seen after application of* t*-BOOH to the spinal cord. In whole-cell patch clamp recordings of spinal dorsal horn neurons, application of* t*-BOOH depolarized membrane potential and increased the frequency of action potential evoked by injection of a current. These results suggest that increases in the superoxide intermediates OH^∙^ and OCl^−^ sensitize dorsal horn neurons and thereby produce hyperalgesia in normal rats.

ROS play a critical role in normal physiological functions and various pathological conditions [2]. The many types of ROS include O_2_^∙−^, OH^∙^, H_2_O_2_, OCl^−^, NO, and peroxynitrite [2, 3]. These ROS have been linked to pathological conditions, including pain. Recently, many ROS scavengers have been shown to induce analgesic effects for neuropathic and inflammatory pain. In several rat models of pain, tirilazad [22], superoxide dismutase mimetics [5], phenyl-N-*tert*-butylnitrone [6, 7], 5,5-dimethyl-1-pyrroline-N-oxide [6], and vitamin E [8] reduced hyperalgesic behaviors, mainly through the action on the spinal cord [6, 8]. Those findings suggest that free radicals in the spinal cord are critically involved in neuropathic and inflammatory pain. However, it is still unclear which types of ROS in the spinal cord are critical for production of pain behaviors.

Superoxide radicals (O_2_^∙−^), a primary ROS formed in cells, facilitate OH^∙^ production by reacting H_2_O_2_ with intracellular iron (i.e., Fenton reaction).* t*-BOOH, a donor of OH^∙^, has been used widely in oxidative stress experiments because it easily penetrates the cell membrane, breaks down to produce ROS as a simple chemical reaction, and is further decomposed into OH^∙^ by increasing the level of free iron in cells [23–25].* t*-BOOH induces spinal long-term potentiation in superficial cord slices, and this long-term potentiation is maintained for 20–30 min after* t*-BOOH is washed out, suggesting the involvement of OH^∙^ [11].* t*-BOOH decreases the frequency of inhibitory postsynaptic currents (IPSCs) without affecting the amplitude of IPSCs [26]. The development of hyperalgesia and enhanced sensitivity of spinal WDR neurons after treatment with* t*-BOOH in our study may have been due to a reduction of inhibitory neurotransmission stemming from an increase of OH^∙^ in the spinal cord.

NaOCl, used as a donor of hypochlorite in this study, hydrolyzes to hypochlorous acid (HOCl) in the solution. HOCl can be naturally produced by chloride ions and H_2_O_2_ in the presence of myeloperoxidase. As microglia, monocytes/macrophages, and neutrophils are a major source for myeloperoxidase [27–29], HOCl can be produced in the nervous tissue. HOCl crosses the plasma membrane, inactivates intracellular enzymes, inhibits mitochondrial respiration, and rapidly oxidizes intracellular glutathione [30–33]. In addition, HOCl can react with a wide range of functional groups, such as thiol, thioether, amino, and heme groups [34, 35]. It can also generate highly reactive singlets and hydroxyl radicals with H_2_O_2_ and O_2_^∙−^ [36, 37]. Moreover, HOCl can react with nitrite, the major end-product of nitric oxide, to form highly reactive nitryl chloride [38]. A previous study showed that hypochlorite (OCl^−^) activates calcium influx and membrane currents via mediation of TRPA1 (transient receptor potential cation A1) channels, contributing to pain behaviors [39]. In the present study, intrathecal injection of NaOCl decreased mechanical thresholds of hind paws. Therefore, induction of hyperalgesia by intrathecal NaOCl is likely caused by activation of sensory neurons in the spinal dorsal horn or conversion of NaOCl into reactive radicals, such as OH^∙^.

In our* in vivo* extracellular recordings, the evoked responses of WDR neurons in the spinal dorsal horn were greatly enhanced by* t*-BOOH treatment, with responses eventually returning to baseline levels. Whole-cell patch clamp recordings revealed that treatment with* t*-BOOH enhanced the excitability of spinal dorsal horn neurons, depolarized the membrane potential of SG neurons, and increased the amplitude and frequency of EPSPs. The EPSP changes in SG neurons began within 2-3 min after application of* t*-BOOH and were maintained for up to 5–30 min after washout. These findings suggest that increased ROS, particularly OH^∙^, in the spinal cord critically contribute to central sensitization via modulation of excitatory neurotransmission.

The functional roles of H_2_O_2_ have been studied extensively in various brain regions. In the brain, H_2_O_2_ influences neuronal excitability by modulating synaptic transmission and the activation of various ion channels. H_2_O_2_ induces hyperexcitability in thalamic neurons by altering the balance between excitatory and inhibitory synapses [40]. It also produces hyperexcitability by activating the N-methyl-D-aspartate receptor [41] and by increasing extracellular glutamate [42, 43] in hippocampal or cortical neurons. In addition, H_2_O_2_ induces membrane depolarization and increases excitability in medium spiny neurons via activation of transient receptor potential channels [44]. In contrast, few studies have examined the role of H_2_O_2_ in spinal dorsal horn neurons in development of hyperalgesia. One study showed that when injected into the plantar surface of hind paws in mice, H_2_O_2_ induced thermal or mechanical hyperalgesia in a dose-dependent manner [45]. In another study, Takahashi et al. [46] reported that H_2_O_2_ increased the frequency of GABAergic miniature inhibitory postsynaptic currents in SG neurons, thereby leading to antihyperalgesia. In the present study, spinal injection of H_2_O_2_ failed to induce hyperalgesia or pain behaviors. Taken together, these findings suggest that H_2_O_2_ in spinal dorsal horn neuron may not participate directly in generation of hyperalgesia.

The recorded SG neurons were classified as AFNs or TFNs, based on their intrinsic firing properties evoked by intracellular current injection [19, 20]. AFNs generate short burst of spikes at the beginning of depolarization and most of them are physiologically classified as nociceptive neuron. TFNs exhibit repetitive spike firing and little adaptation during sustained depolarization and majority of them are shown to be wide dynamic range (WDR) or nociceptive neurons [20]. Superfusion of* t*-BOOH increased the firing frequency of TFNs but not AFNs in our study, indicating that* t*-BOOH treatment increased the excitability of WDR or nociceptive neurons. In combination with the data from our* in vivo* extracellular recordings showing that intrathecal administration of* t*-BOOH caused enhanced excitability of WDR neurons in normal rats, the results suggest that ROS, particularly OH^∙^, generate hyperalgesia by acting on TFNs or other WDR neurons.

## 5. Conclusion

Intrathecal administration of the ROS donors* t*-BOOH and NaOCl significantly decreased the mechanical thresholds of pain behaviors and increased the responses of WDR neurons in the spinal dorsal horn to mechanical stimuli in normal rats.* t*-BOOH also increased the membrane excitability of SG neurons in spinal cord slices. Our findings indicate that ROS, particularly OH^∙^, play an important role in the development of pain and the process of central sensitization in the spinal cord. Reducing the levels of ROS in the spinal cord may therefore be an effective way to treat pain.
